# Stabilization of Ribosomal RNA of the Small Subunit by Spermidine in *Staphylococcus aureus*

**DOI:** 10.3389/fmolb.2021.738752

**Published:** 2021-11-18

**Authors:** Margarita Belinite, Iskander Khusainov, Heddy Soufari, Stefano Marzi, Pascale Romby, Marat Yusupov, Yaser Hashem

**Affiliations:** ^1^ Institut de Génétique et de Biologie Moléculaire et Cellulaire (IGBMC), INSERM U964, CNRS UMR7104, Université de Strasbourg, Illkirch, France; ^2^ Architecture et Réactivité de l’ARN, CNRS 9002, Université de Strasbourg, Strasbourg, France; ^3^ Institut Européen de Chimie et Biologie (IECB), ARNA U1212, Université de Bordeaux, Pessac, France; ^4^ Institute of Fundamental Medicine and Biology, Kazan Federal University, Kazan, Russia

**Keywords:** ribosome 70 S, ribosomal RNA, *Staphylococcus aureus*, translation, RNA stability

## Abstract

Cryo-electron microscopy is now used as a method of choice in structural biology for studying protein synthesis, a process mediated by the ribosome machinery. In order to achieve high-resolution structures using this approach, one needs to obtain homogeneous and stable samples, which requires optimization of ribosome purification in a species-dependent manner. This is especially critical for the bacterial small ribosomal subunit that tends to be unstable in the absence of ligands. Here, we report a protocol for purification of stable 30 S from the Gram-positive bacterium *Staphylococcus aureus* and its cryo-EM structures: in presence of spermidine at a resolution ranging between 3.4 and 3.6 Å and in its absence at 5.3 Å. Using biochemical characterization and cryo-EM, we demonstrate the importance of spermidine for stabilization of the 30 S *via* preserving favorable conformation of the helix 44.

## Introduction

Protein synthesis is a tightly regulated biological process performed by the ribosome. High-resolution structures of the ribosome and its functional complexes led to major advances in understanding the functioning and the dynamics of this complex machinery (Javed and Orlova, 2019). The bacterial ribosome (70 S) can be divided into the large (50 S) and the small (30 S) subunits. The latter, which contains the decoding center, consists of 21 proteins (r-proteins) and of the 16 S RNA (rRNA). This flexible subunit faces several conformational changes during translation (Frank et al., 2007; Agirrezabala et al., 2008; Zhang et al., 2009) and its stability is often influenced by ions conditions, making the structural analysis challenging.

The 30 S ribosomal subunit is the main platform, where mRNA and initiator P-tRNA are positioned during translation initiation, as well as accommodation of incoming A-tRNA during the elongation stage. During translation initiation, it interacts with the three Initiation Factors (IFs) and undergoes several rearrangements of the head (swiveling/nodding), which affect the mRNA channel (Hussain et al., 2016). Furthermore, each cycle of tRNA translocation through the ribosome is also accompanied by the reversible rotation of the whole 30 S subunit and swiveling of its head (Ogle et al., 2001; Yusupova et al., 2001; Selmer et al., 2006a; Berk et al., 2006; Pisarev et al., 2008; Demeshkina et al., 2010; Jenner et al., 2010; Guo and Noller, 2012; Pulk and Cate, 2013). The body of the 30 S subunit seems to be less affected by these conformational changes (Hussain et al., 2016). Helix 44 (h44) of the 16S rRNA is among the most crucial regions of the 30 S body because it is involved in several bridges with the 50 S (Yusupov et al., 2001; Schuwirth et al., 2005; Selmer et al., 2006b), it interacts with both IF1 and IF3, and is part of the decoding center of the ribosome forming the P-site and being involved in the accommodation of tRNA at the A-site (Demeshkina et al., 2012; Rozov et al., 2015; Rozov et al., 2016; Rozov et al., 2019). Its structure needs to be stable enough to be maintained during translation elongation and sufficiently flexible to allow mRNA and factors binding during translation initiation. For these reasons, several proteins (e.g., Era, RbfA, RimM), which are involved in the ribosome biogenesis, perform quality control function of the h44 as one of the last checkpoints of the 16S rRNA maturation (Dammel and Noller, 1995; Bylund et al., 1998; Datta et al., 2007; Guo et al., 2013; Razi et al., 2019; Schedlbauer et al., 2020). Altogether, for efficient participation of the 30 S in protein synthesis, flexibility and structural integrity are required at the same time, and especially proper folding of h44 is essential.

Recent study has shown the importance of magnesium ions on structure stability of the 30 S from *E. coli* (Jahagirdar et al., 2020). Moreover, numerous studies have led to the conclusion that polyamines, which are present in all types of cells, can stabilize the structure of the ribosome (Zillig et al., 1959; Cohen and Lichtenstein, 1960; Stevens, 1969; Weiss and Morris, 1970; Cohen, 1971; Hardy and Turnock, 1971; Turnock and Birch, 1973). It was shown that *E. coli* cells grown in the absence of polyamines contained a large portion of defective 30 S particles (Echandi and Algranati, 1975). Furthermore, polyamines stimulate the assembly of 30 S ribosomal subunits and thereby increase general protein synthesis rate 1.5- to 2.0-fold (Echandi and Algranati, 1975; Igarashi et al., 1980; Igarashi and Kashiwagi, 2018).

In this study, we solved cryo-EM structures of *S. aureus* 30 S subunit bound to *S. aureus*-specific *spa* mRNA that encodes a virulence factor protein A. We show how the addition of spermidine helps to improve the resolution from 5.3 Å to 3.4 Å (for the SSU body) and 3.6 Å (for the SSU head). The main effect of spermidine was on h44 that presents its active conformation only when the polyamine was added. Under these conditions, the 30 S adopts a closed conformation where the decoding channel is properly formed, and the mRNA is naturally adapted inside the channel. Our work highlights the importance of polyamines in determining the structure of the 30 S subunits by cryo-EM and could be even relevant for functional studies. The protocol for 30 S purification can be easily applied for the preparation of various functional complexes of *S. aureus*.

## Materials and Methods

### 70 S Ribosome Purification

The protocol described in the article Khusainov et al. (2016b) was used for 70 S purification (Khusainov et al., 2016a). *S. aureus* cells (RN6390 strain) were grown at 37°C (180 rpm) in brain-heart infusion broth (BHI) and harvested in the early logarithmic phase (1 OD^600^ / ml). Then cells were washed in buffer A (20 mM Hepes-KOH pH 7.5, 100 mM NH_4_Cl, 21 mM Mg(OAc)_2_, 1 mM DTT), pelleted at 4,750 g and kept frozen at −80^o^C. Typically, 5 g of cells were obtained from 2 L of culture. Lysis of the cells (5 g) was performed in buffer A in the presence of 1 mM EDTA, lysostaphin (Sigma-Aldrich), DNase I (Roche), and protease inhibitor cocktail (Roche) at 37^°^C for 45 min followed by centrifugation at 30,000 g for 90 min.

Ribosomes were precipitated through two stages by adding to the supernatant PEG 20,000 (Hampton Research) with final concentrations of 2.8 and 4.2 % w/v. Solutions were centrifuged at 20,000 g for 5 and 10 min, respectively. The pellet was then resuspended in 35 ml of buffer A and layered to 25 ml cushion of buffer B (10 mM Hepes-KOH pH 7.5, 500 mM KCl, 25 mM Mg(OAc)_2_, 1.1 M Sucrose, 0.5 mM EDTA, 1 mM DTT), followed by centrifugation at 158,420 g for 15 h using a Beckman Type 45 Ti rotor.

The ribosomal pellet from sucrose cushion was resuspended in buffer E (10 mM Hepes-KOH pH 7.5, 100 mM KCl, 10.5 mM Mg(OAc)_2_, 0.5 mM EDTA, 1 mM DTT), and loaded on 7–30 % w/v sucrose gradients at a concentration up to 7 mg/ ml and centrifuged at 38,694 g for 15.5 h using a Beckman SW28 rotor. Magnesium was adjusted to 25 mM in the pooled fractions. Ribosomes were precipitated by adding PEG 20,000 to a final concentration 4.5 % w/v and centrifuged at 20,000 g for 12 min. The pellet was gently resuspended in buffer G (10 mM Hepes-KOH pH 7.5, 30 mM NH_4_Cl, 10 mM Mg(OAc)_2_, 1 mM DTT), flash-frozen in liquid nitrogen and stored at −80^o^C in small aliquots.

### Effect of Ionic Conditions on the 30 S Ribosomal Subunit Purification

**FIGURE 1:**
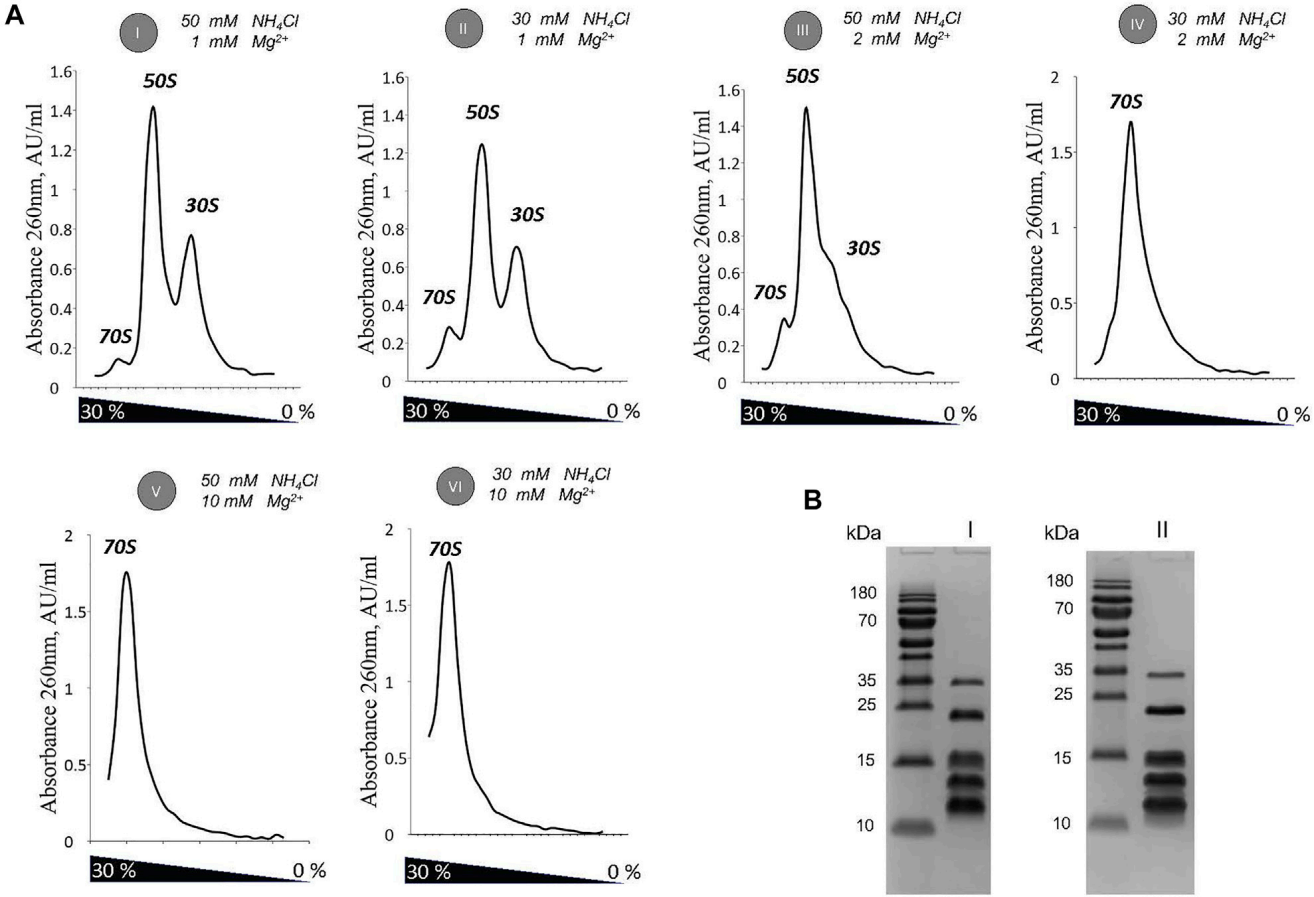
Optimization of 30 S subunit purification by titration of Mg^2+^ / NH_4_Cl ratio. (**A)** Sucrose gradients profiles of the 70 S sample exposed to mild dissociation conditions (I–IV), and association conditions (V-VI). **(B)** SDS-PAGE of 30 S purified at conditions determined as optimal (1 mM Mg / 30 mM NH_4_Cl) in absence (I) and presence (II) of spermidine.

The small ribosomal subunit was isolated from the intact 70 S ribosome under various conditions described below. First, the 70 S ribosome was dialysed in dissociation buffer (1 mM Mg(OAc)_2_, 200 mM NaCl_2_ or 200 mM KCl, 10 mM Hepes-KOH pH7.5, 1 mM DTT) for 4 h at 4^°^C. Then, the subunits were separated with 0–30 % w/v sucrose gradients followed by centrifugation at 35,606 g for 17 h at 4^o^C using Beckman SW28 rotor. Fractionation of the sucrose gradient is shown on Supplementary Figure S1A,B. Fractions from the sucrose gradient in 200 mM KCl were pooled and loaded again on 5–20 % w/v sucrose gradients (35,606 g for 17 h, Beckman SW28 rotor, Supplementary Figure S1C). The search for optimal salt conditions for 30 S purification was carried out in 100 and 200 mM KCl or NH_4_Cl (1 mM Mg(OAc)_2_, 10 mM Hepes-KOH pH7.5, 1 mM DTT) and 400 mM NaCl (6 mM or 10 mM Mg(OAc)_2_, 10 mM Hepes-KOH pH7.5, 1 mM DTT). Dialysis for 4 h at 4^o^C was followed by subunits separation step with 0–30 % w/v sucrose gradient and centrifuged at 44,556 g for 15 h using Beckman SW41 rotor. Selected fractions were pooled, concentrated with Amicon 0.5 ml MWCO 100K, and analyzed on a 15 % SDS-PAGE (Supplementary Figures S2A,B). Re-screening for optimal conditions was performed in 30 and 50 mM NH_4_Cl (1 mM Mg(OAc)_2_, 10 mM Hepes-KOH pH7.5, 1 mM DTT). The 70S ribosome was divided into subunits with 0–30 % w/v sucrose gradient followed by centrifugation at 46,932 g for 14 h 14 min using Beckman SW41 rotor. The obtained sucrose gradient profiles and respective SDS-PAGE profiles of pooled 30 S peak samples are shown in Figures 1A,B.

### 30 S Ribosomal Subunits Purification

For structural analysis of the 30 S subunit, we first purified 70 S particles as described above, with tiny modification. At the last step, the 70 S ribosomes were dissolved in buffer G’ (10 mM Hepes-KOH pH 7.5, 30 mM NH_4_Cl, 1 mM Mg(OAc)_2_, 1 mM DTT) omitting the freezing step ribosomes were directly loaded on 0–30 % w/v sucrose gradients equilibrated in buffer G’ and centrifuged at 61,739 g for 14.5 h using a Beckman SW28 rotor. The concentration of Mg(OAc)_2_ was adjusted to 10 mM in the selected pooled fractions. In addition, the sample was supplemented with 2.5 mM of spermidine and concentrated using 100K Amicon ultra centrifugal filters (Merck Millipore). Aliquots were flash-frozen in liquid nitrogen and stored at −80°C.

### mRNA Purification

*S. aureus spa* mRNA, encoding protein A, has UUG start codon and a strong Shine and Dalgarno (SD) sequence AGGGG. The sequence of the full-length *spa* mRNA used in the study is shown in Supplementary Figure S2C. The RNA was transcribed from a plasmid as previously described in (Benito et al., 2000; Huntzinger et al., 2005). In brief, the plasmid was linearized by Bam*HI* during 2 h at 37°C, and the mRNA was *in vitro* transcribed during 3 h at 37°C with T7 RNA polymerase. The mRNA was separated on a 6 % polyacrylamide midi-sized gel and eluted in a solution containing 16 % phenol pH 4.5–5 with 50 mM ammonium acetate and 1 mM EDTA.

### *In vitro* Reconstruction of 30 S With *spa* mRNA

The 30 S ribosomal subunits were incubated with *spa* mRNA during 15 min at 37°C in the buffer containing 10 mM Hepes-KOH pH 7.5, 30 mM NH_4_Cl, 10 mM Mg(OAc)_2_ in the presence or absence of 2.5 mM spermidine.

### Cryo-EM Data Acquisition

4 µL of samples (containing the 30 S bound to mRNA) at 90 nM was applied on Quantifoil R2/2300-mesh holey carbon grids covered with carbon in a temperature- and humidity-controlled Vitrobot Mark IV (T = 4°C, humidity 100%, blotting time 2 s, blotting force 5, waiting time 30 s). The data acquisitions were performed on a Talos Arсtica instrument (FEI Company) at 200 kV using the EPU software on the Falcon three direct detector device (FEI Company). Data were collected at a nominal under focus of −0.5 to −2.7 μm at a magnification of 120,000 X yielding a pixel size of 1.24 Å.

### Electron Microscopy Image Processing

Drift and gain correction, and dose weighting were performed using MotionCor2 (Zheng et al., 2017). A dose weighted average image of the whole stack was used to determine the contrast transfer function with the software Gctf (Zhang, 2016). The following process has been achieved using RELION 3.0 (Zivanov et al., 2018). Particles were picked using a Laplacian of Gaussian function (min diameter 180 Å, max diameter 290 Å). For the 30S-mRNA complex without polyamine, after 2D classification, 256,000 particles were extracted with a box size of 248 pixels and binned four folds for 3D classification into five classes (final = 5.3 Å resolution). For the 30−S-mRNA complex with spermidine, after 2D classification, 529,602 particles were extracted with a box size of 270 pixels and binned three-fold for 3D classification into six classes. Three classes depicting high-resolution features have been selected for refinement. The obtained structure has been refined up to 3.6 Å resolution. Individual focused refinement of the head and the body of the small ribosomal subunit led to the resolution 3.6 Å and 3.4 Å respectively (Supplementary Figures S3A,B). Local resolution estimation was performed in RELION 3.0 using RELION implementation and visualized in Chimera using Surface color option (Supplementary Figure S3C).

### Structure Building and Model Refinement

As the initial model, we used 30−S extracted from the *S. aureus* vacant 70S ribosome (PBD 5LI0 [https://doi.org/10.2210/pdb5LI0/pdb]). The initial coarse fitting was performed using the NAMDinator web service (Kidmose et al., 2019), which implements the algorithms of molecular dynamics flexible fitting (MDFF) (Trabuco et al., 2009). The default parameters that were used for flexible fitting (start temperature = 298 K; G-force scaling factor = 0.3; minimization steps = 2000; simulation steps = 20,000). Then the real-space refinement was performed in PHENIX (Afonine et al., 2018) (starting temperature = 800 K; cool rate = 100 K). Ribosomal RNA was corrected in ERRASER web service (Chou et al., 2013), which uses enumerative real-space refinement assisted by electron density under Rosetta protocol. Obtained model was corrected manually in Coot (Supplementary Figure S5); (Emsley and Cowtan, 2004). The model validation was done in MolProbity web service (Chen et al., 2010). Figures featuring cryo-EM densities as well as atomic models were visualized with UCSF Chimera (Pettersen et al., 2004) and ChimeraX (Goddard et al., 2018). The coordinates of the head and the body parts were rigid body fitted into the 30S-mRNA with spermidine density map to build the full 30 S model followed by manual curation in coot.

## Results

### Optimization of Salt Conditions for the 30 S Purification

To analyze the effect of ionic conditions on stability of the 30 S subunit, we performed sucrose gradient sedimentation assays of the 70 S ribosome equilibrated in H_10_K_200_M_1_ (10 mM Hepes-KOH pH 7.5 (at 25^o^C), 200 mM KCl, 1 mM Mg(OAc)_2_) or H_10_Na_200_M_1_ (10 mM Hepes-KOH pH 7.5 (at 25^o^C), 200 mM NaCl, 1 mM Mg(OAc)_2_) buffers. The ribosomes sedimented in H_10_K_200_M_1_ buffer dissociated into 50 S and 30 S subunits (Supplementary Figure S1A). Conversely, in presence of 200 mM NaCl, the dissociation of the 70 S ribosomes into the two subunits was not observed (Supplementary Figure S1A). However, the shift of the peak towards light fractions of the gradient and the peak asymmetry suggested the loss of structural integrity of these subunits (Supplementary Figure S1B). To further monitor the integrity of the dissociated ribosomal subunits, the 50 S and 30 S peaks were loaded onto sucrose gradients equilibrated in a buffer containing 200 mM KCl. A bifurcation of the 30 S peak was detected with the appearance of particles with a lower molecular weight than 30 S (Supplementary Figure S1C, right panel) suggesting the inability of the small subunit to withstand 200 mM KCl for a prolonged period of time.

Therefore, we repeated experiments with amended ionic conditions using 100 and 200 mM of NH_4_Cl or KCl, respectively at constant 1 mM magnesium acetate. Other conditions included 400 mM NaCl with either six or 10 mM magnesium acetate. For each experiment, the content of the ribosomal proteins within the 30 S peak was analyzed using SDS-PAGE analysis. Despite similarities in the sucrose gradient profiles (Supplementary Figure S1A), higher salt concentrations, especially with 200 mM KCl or 400 mM NaCl, led to the loss of several ribosomal proteins (Supplementary Figure S1B). Taken together, all further experiments were conducted using NH_4_Cl, which has the least dissociating effect on the binding of several ribosomal proteins.

After performing a fine analysis of Mg/NH_4_Cl balance effect on ribosome dissociation, we selected 1 mM Mg^2+^ in combination with 30 mM NH_4_Cl to isolate 30 S from 70S for further cryo-EM analysis (Figures 1A,B). These conditions provided an efficient dissociation of the 70 S ribosome into subunits and concomitantly had their mildest effect on the loss of ribosomal proteins.

### Spermidine Effect on the Helix 44 and mRNA Positioning

Using optimized salt concentrations for 30 S purification, we solved the cryo-EM structure of the 30 S in complex with *S. aureus spa* mRNA at a resolution of 3.6 Å. The complex was formed in H_10_NH_30_M_10_ (10mM Hepes-KOH pH7.5 (at 25^o^C), 30mM NH_4_Cl, 10 mM Mg(OAc)_2_ and 1mM DTT). Initially, two different datasets were collected in presence (dataset 2) and in absence (dataset 1) of spermidine.

**FIGURE 2:**
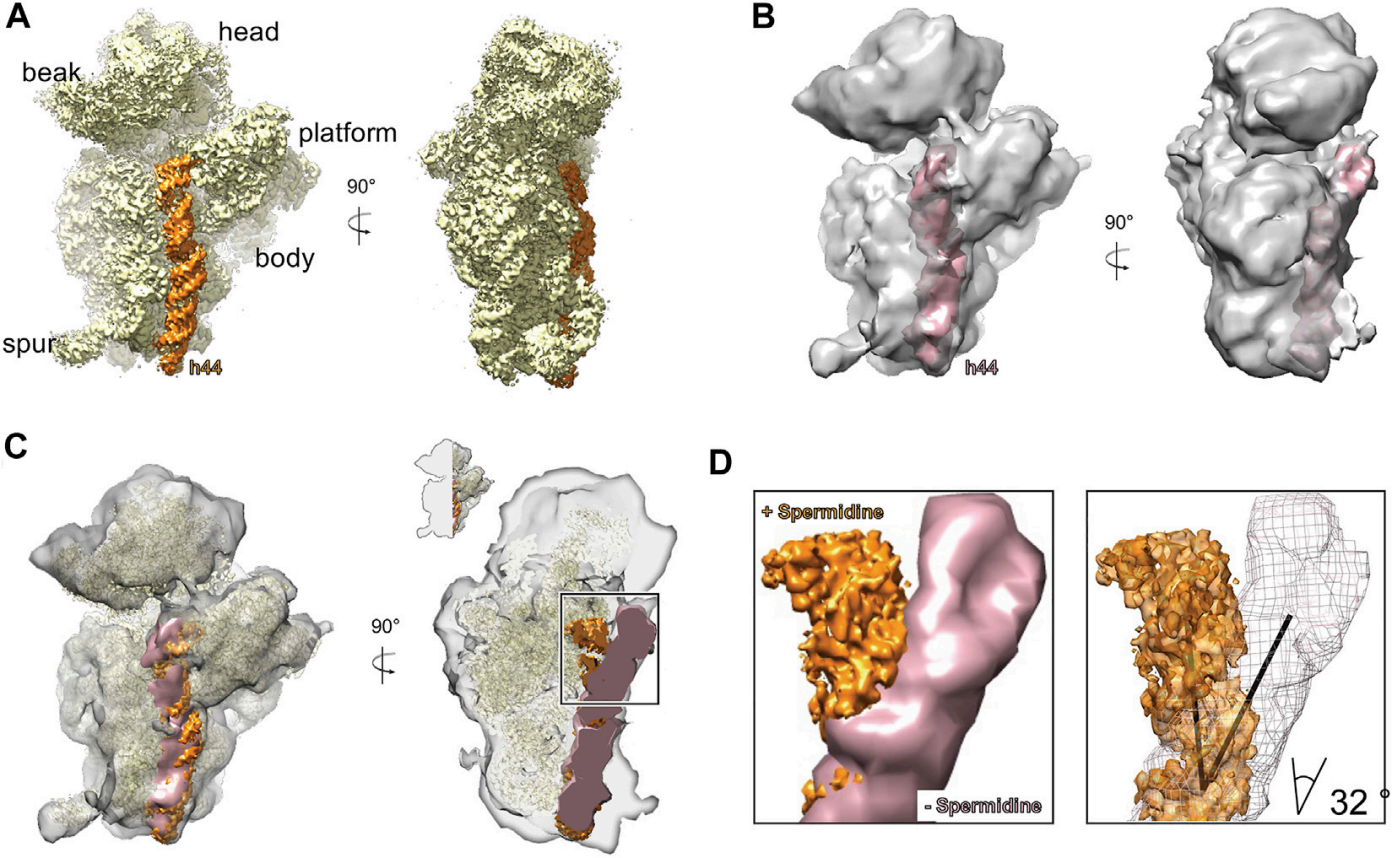
Addition of spermidine stabilizes helix 44 of the 30 S. A. An overview of the structure of 30 S subunit prepared with addition of 2.5 mM spermidine **(A)** and without spermidine **(B)**. The h44 is highlighted in orange and pink respectively. **(C)** An overlay of the structures showing that without spermidine, h44 is destabilized and bent away from the head. For the A-site view (right) structures were clipped as indicated in the inset. **(D)** A close-up view on h44 conformation in 30S with addition of spermidine (orange) and without it (pink/mesh). The calculated angle of the tilt is 32°.

*In silico* sorting the complex lacking spermidine was divided into five classes (Supplementary Figure S3). In classes 2, 3, and 4, the upper part of h44 near the decoding center (nucleotides 1,414–1,431 and 1,490–1,508) was tilted by about 32°, whereas the lower part of h44 (1,430–1,489) was only partially visible (Figures 2B–D). In addition, in classes 1, 2, and 3, only partial densities of uS2 and bS6 proteins were detected (Supplementary Figure S4).

In the presence of spermidine, h44 acquired more stable conformation (Figure 2A). It is also worth noting that all small subunit ribosomal proteins except bS21 were present in the structures. Indeed, the density of bS21 protein was not identified due to the positioning of *spa* mRNA in the region where this protein was previously determined (Korostelev et al., 2007; Khusainov et al., 2017). In addition, in both datasets, a high concentration of Mg^2+^ was used for complex formation, which allows us to conclude that polyamines and magnesium ions at these concentrations have different effects on obtained structures.

**FIGURE 3:**
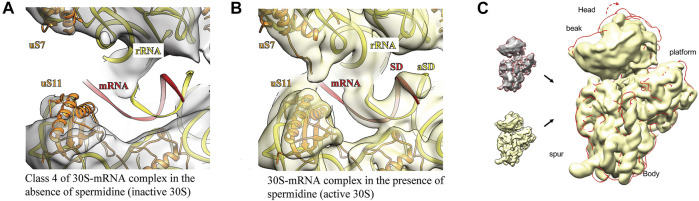
Addition of spermidine helps to close the mRNA channel, allowing mRNA to fully accommodate in it. The ribosomal RNA is highlighted in yellow, ribosomal proteins–in orange and messenger RNA–in red. For the panel **(A)** the same atomic model was used as for panel **(B)** with additional processing in NAMDinator and refitting head and body parts separately. Panel **(C)** represents overlay of the 30 S-mRNA structures in the absence (grey + red contour) and the presence of spermidine (yellow). The 30 S head adopts open conformation (red contour, red arrow) in the absence of spermidine.

Open head conformation was observed in all 3D classes obtained from polyamine-deficient 30 S particles; however, mRNA was barely discernible only in class 4 (Figure 3A). When spermidine was added, the mRNA channel was closed by rotation of the 30 S head, allowing *spa* mRNA to fully accommodate in the channel (Figures 3B,C) similarly to the 70S·mRNA·fMet-tRNA^fMet^ complex (Golubev et al., 2020). The well-defined density found in the Shine-Dalgarno-anti-Shine-Dalgarno (SD:aSD) region allowed us to fairly accurately build the 30S-mRNA model in this region.

## Discussion

In this work, we describe the protocol for obtaining the structure of an intact and homogenous 30 S ribosomal subunit from *Staphylococcus aureus*. We demonstrate a strong dependency of 30 S stability upon ionic conditions during purification. This effect is observed in species-dependent manner. *S. aureus* 30 S showed the inability to withstand 200 mM NaCl concentration and an increased sensitivity to KCl in the same range of concentration (Supplementary Figures S1A–C). This is different to *Thermus thermophilus or Escherichia coli* ribosomes that can maintain their structure under similar ionic conditions, even in combination with reverse phase chromatography performed in up to 1.5 M ammonium sulfate concentration (Kirillov et al., 1978; Trakhanov et al., 1987; Clemons et al., 2001). Interestingly, the early studies of the ribosome from *Bacillus subtilis* also suggest an increased stability in lower concentrations of salt (Fahnestock, 1977). This correlates with our findings and suggests that ribosomes from Gram-positive bacteria have evolved different properties (Figure 1A).

The biochemical characterization of the 30 S ribosomal subunit revealed favourable ionic conditions to avoid 30 S particle distortions during purification [e.g., changes in sedimentation coefficient (Supplementary Figure S1) or loss of ribosomal proteins (Supplementary Figure S2B)]. Further, using cryo-EM we showed that even in optimized salts concentration 30S particles show structural disintegration that can be avoided by addition of spermidine. We have shown that the presence of spermidine is crucial for maintaining the correct folding of the helix (h44) of 16S rRNA near the decoding center (Figure 2). Spermidine is a polyamine known to stabilize the folding of RNA molecules including rRNA (Cohen and Lichtenstein, 1960; Weiss and Morris, 1970; Cohen, 1971; Hardy and Turnock, 1971; Turnock and Birch, 1973). The upper part of h44 was found as one of the preferred sites for the binding of polyamines (Amarantos et al., 2002) and was particularly observed in the 70S crystal structure from *E. coli* (Noeske et al., 2015). Although polyamines like spermine and putrescine were used in these studies, the binding sites are believed to be identical for spermidine too. Additionally, nucleotides G931, A1400, C1411 of the 16S rRNA were shown to be polyamine-binding sites (Amarantos et al., 2002). Therefore, it is reasonable to suggest that polyamine molecules bound to those positions may control changes in the head conformation.

Interestingly, polyamines were shown to positively affect protein synthesis depending on the uracil content of mRNAs (Igarashi et al., 1975), and spermidine enhanced translation of those mRNAs carrying the less effective UUG initiation codon (Igarashi and Kashiwagi, 2018). These data suggested that at least some mRNAs require the presence of spermidine *in vitro*. Noteworthy, the *spa* mRNA that we used in this study is a *S. aureus* specific mRNA with UUG start-codon. It is unclear whether spermidine can also facilitate the SD:aSD positioning within the mRNA exit tunnel. Even though our structure in the presence of spermidine has a stable SD:aSD interaction, it may be caused by the general effect on ribosome stabilization rather than direct association of polyamine molecules in this region. In *S. aureus spa* mRNA, Shine-Dalgarno sequence is located in the unstructured region that enables its direct binding to the ribosome in the absence of polyamines (Khusainov et al., 2016b). However, many other mRNAs in *S. aureus* have their SD hidden in the step loop. Speculatively spermidine may facilitate the association of such mRNAs with the ribosome through stabilization of SD:aSD interaction.

Spermidine and Mg^2+^ usually bind similarly to the double-stranded regions of rRNA (Igarashi et al., 1982). Here, we have increased the concentration of Mg^2+^ ions from 1 to 10 mM immediately after the dissociation of the subunits and kept it for the complex formation with *spa* mRNA. However, only the addition of spermidine was able to attain the formation of a functional mRNA-30S binary complex. This is well correlated with previous studies showing that polyamines cannot be compensated by Mg^2+^, and conversely replacement of Mg^2+^ by spermidine leads to a loss of ability to support peptide synthesis (Weiss and Morris, 1970; Teraoka and Tanaka, 1973). Thus, our study provides additional evidence that spermidine and Mg^2+^ are not equivalent in their ability to stabilize the structure of *S. aureus* ribosome.

It was described that helix 44 acquires unfavorable conformation as the result of the absence of several ribosomal proteins located at the interface of the 30 S subunit like uS5, uS12, and bS20 (Supplementary Figure S6). However, these ribosomal proteins are present in our structure solved with or without spermidine. It is also plausible that h44 was deformed as an indirect consequence resulting from the flexibility of uS2, bS6, uS7, and uS11 in the absence of spermidine, or also upon exposing of the 30 S interface to the solvent. Similar to our recent observation, destabilization of some regions of 23S rRNA takes place at the interface of the individual 50 S subunit from *S. aureus* in the absence of 30 S counterpart (Khusainov et al., 2020). Conformational changes in important functional motifs on the platform and at the decoding center can also be caused by maturation factors such as RimM, RbfA, and Era (Dammel and Noller, 1995; Bylund et al., 1998; Guo et al., 2013; Razi et al., 2019; Schedlbauer et al., 2020). The obtained structures showed that the binding sites of the maturation factors to the small ribosomal subunit are located in the upper part of h44 (Datta et al., 2007). This may indicate the need for stabilization of h44 at later stages. It remains to be studied whether such conserved maturation factors are also required at a later step of the 30 S ribosomal subunit assembly in *S. aureus*. Interestingly, deletion of *era* in *S. aureus* caused a strong decrease in 70S formation linked to a defect of 30 S processing (Wood et al., 2019).

In conclusion, our data reveals the requirement to maintain particular ionic conditions and the addition of spermidine during *S. aureus* 30 S ribosomal subunit purification. The described protocol can now be used to solve other functional ribosomal complexes in order to better decipher the differences existed between Gram-positive and Gram-negative bacteria at the initiation step of protein synthesis.

## Data Availability

The datasets presented in this study can be found in online repositories. The names of the repository/repositories and accession number(s) can be found below: http://www.wwpdb.org/, 7KWG; http://www.wwpdb.org/, 7BGD; http://www.wwpdb.org/, 7BGE; https://www.ebi.ac.uk/pdbe/emdb/, EMD-23052; https://www.ebi.ac.uk/pdbe/emdb/, EMD-12178; https://www.ebi.ac.uk/pdbe/emdb/, EMD-12179; https://www.ebi.ac.uk/pdbe/emdb/, EMD-12091; https://www.ebi.ac.uk/pdbe/emdb/, EMD-12090.
