# Supplementary figures and images for: Stabilization of Ribosomal RNA of the Small Subunit by Spermidine in Staphylococcus aureus
Source: Front Mol Biosci. 2021 Nov 18;8:738752. doi: 10.3389/fmolb.2021.738752 (PMC8637172; doi:10.3389/fmolb.2021.738752)

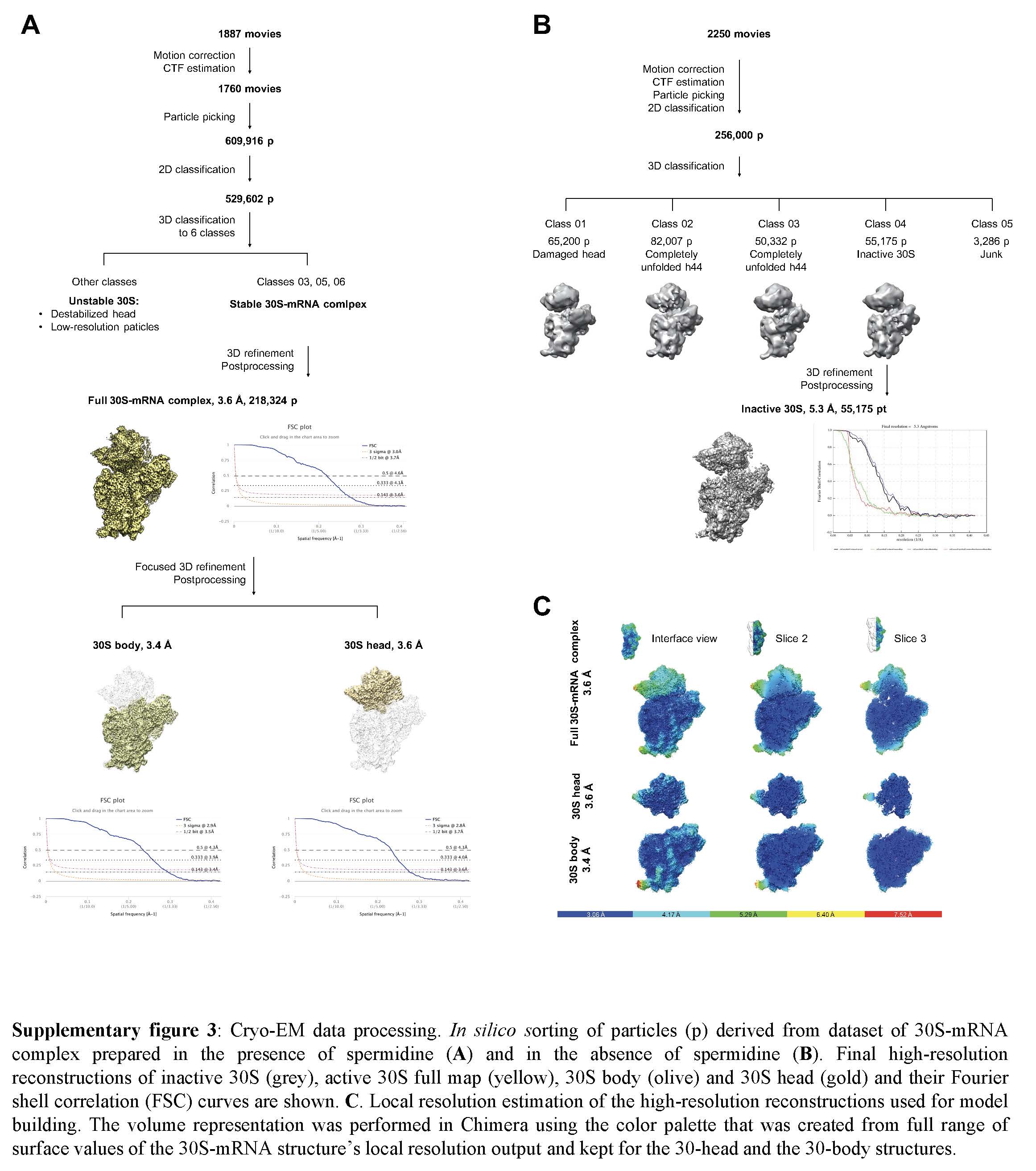

Supplement: Supplementary file 1 [file Image3.JPEG]

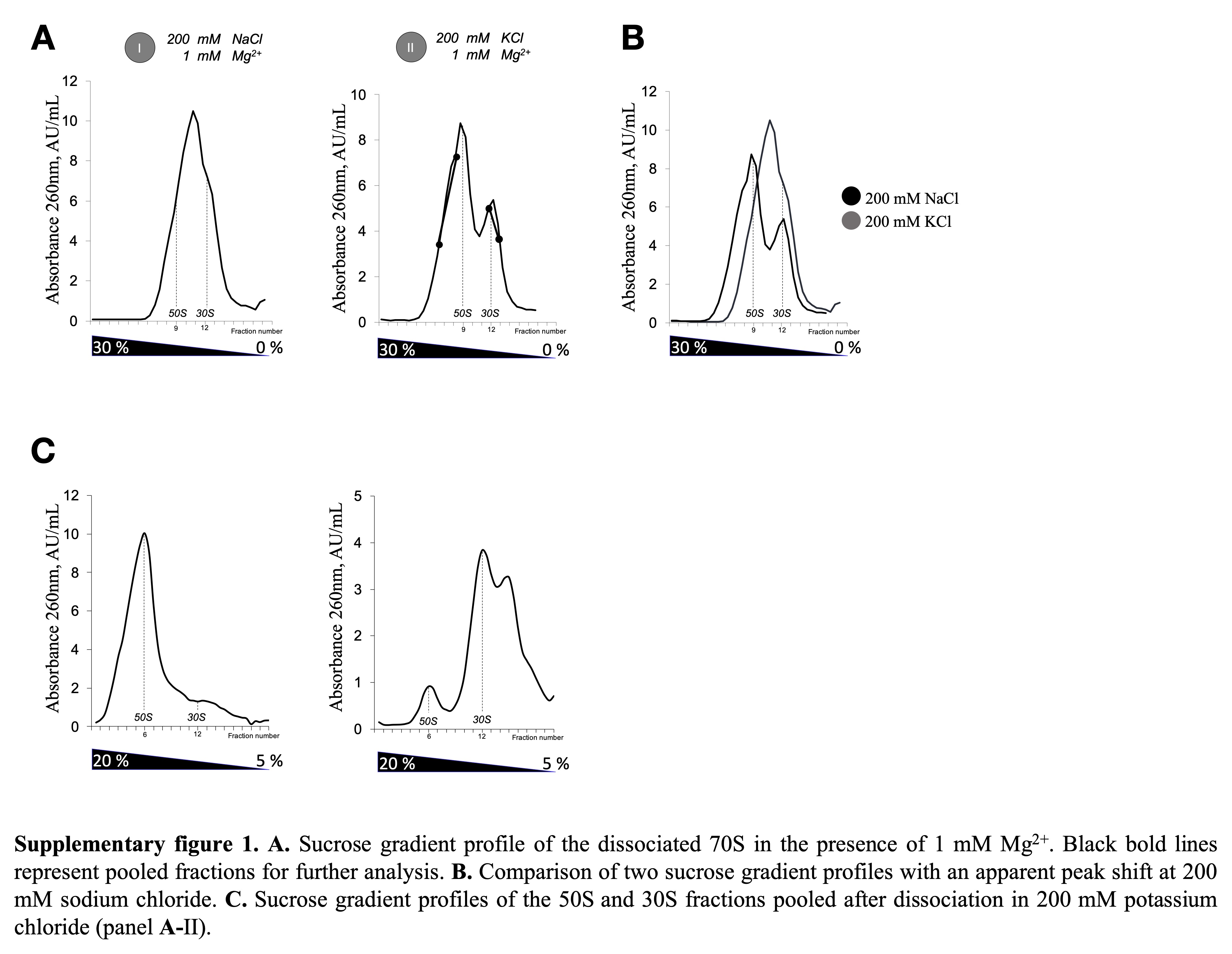

Supplement: Supplementary file 2 [file Image1.JPEG]

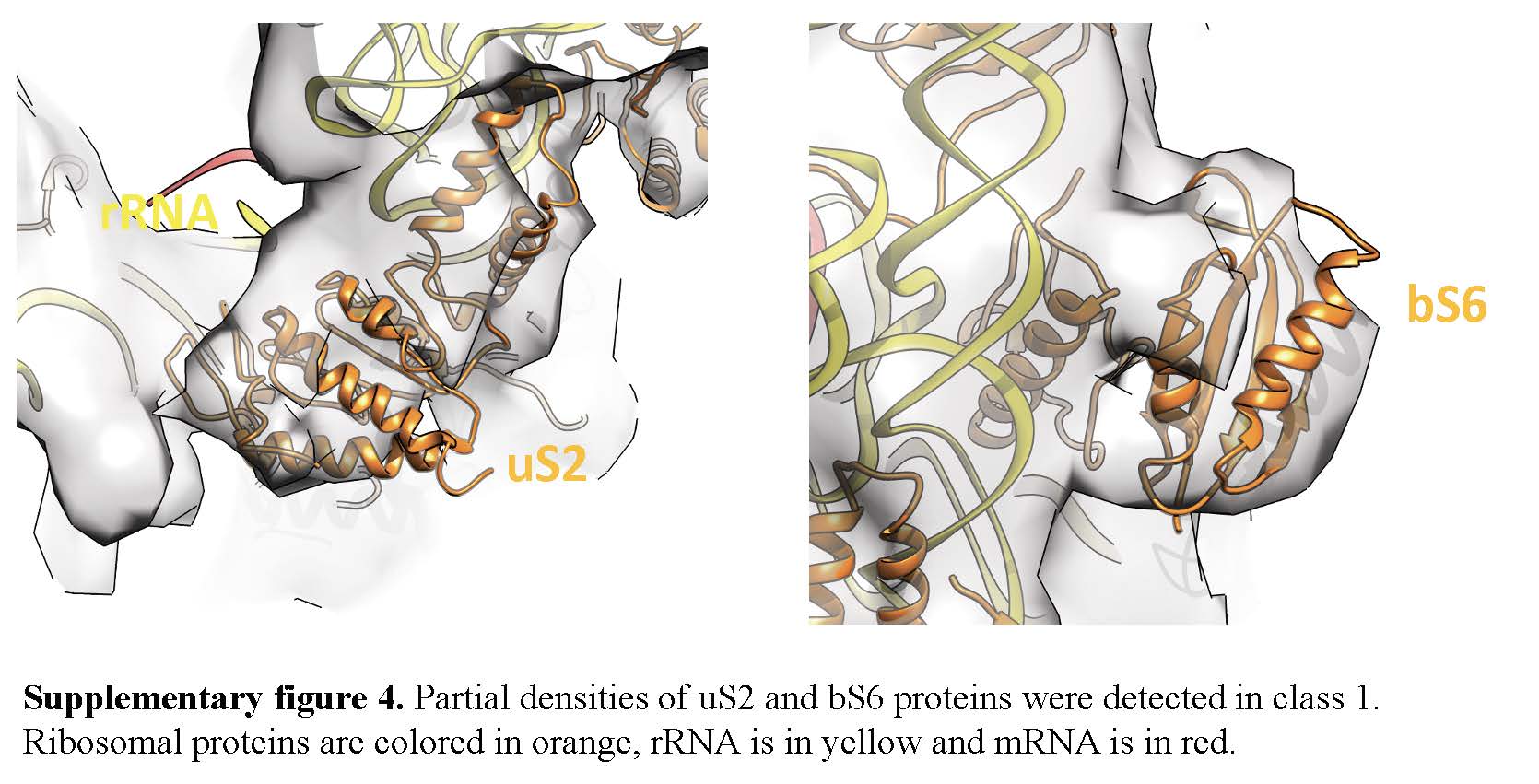

Supplement: Supplementary file 3 [file Image4.JPEG]

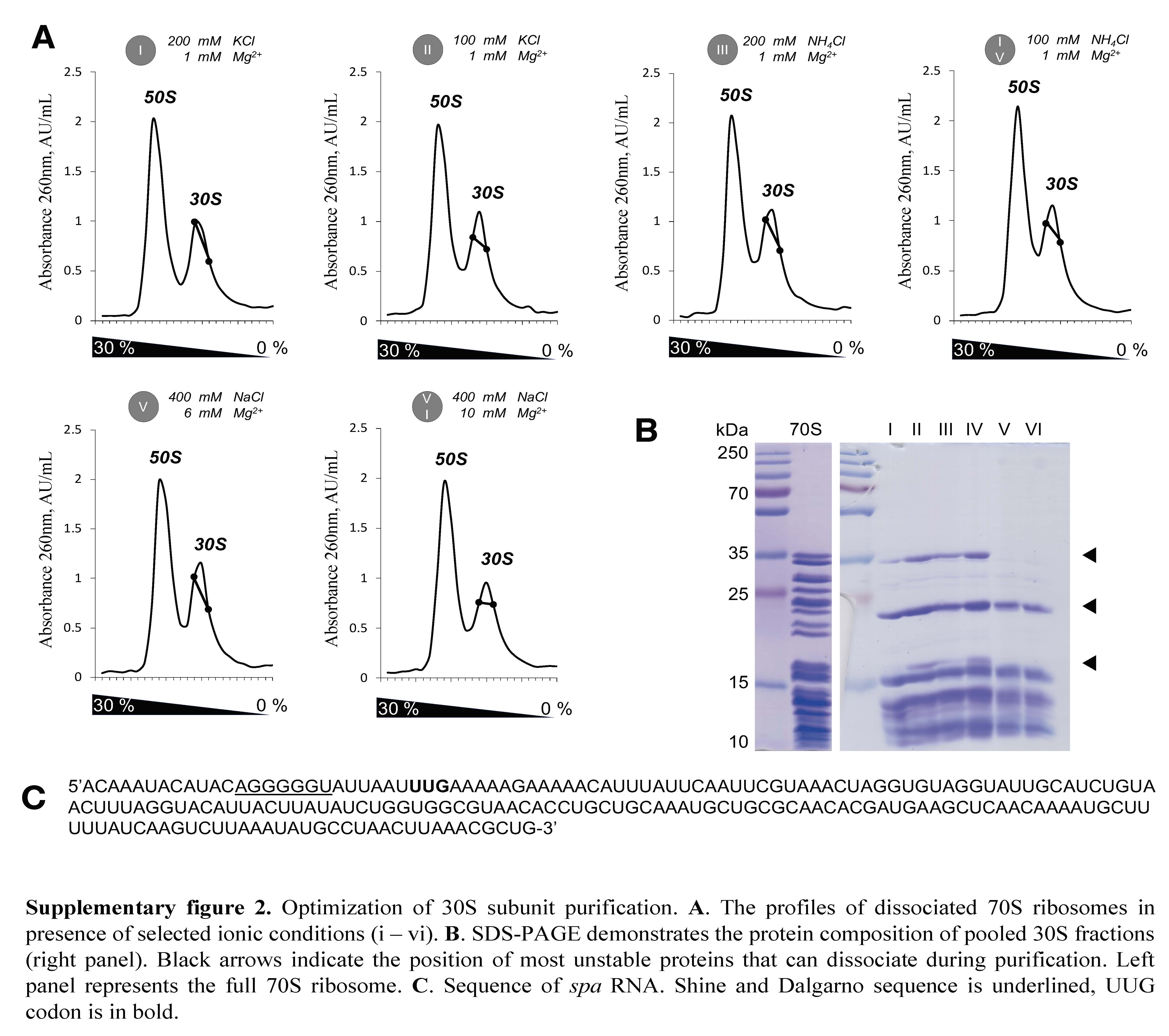

Supplement: Supplementary file 4 [file Image2.JPEG]

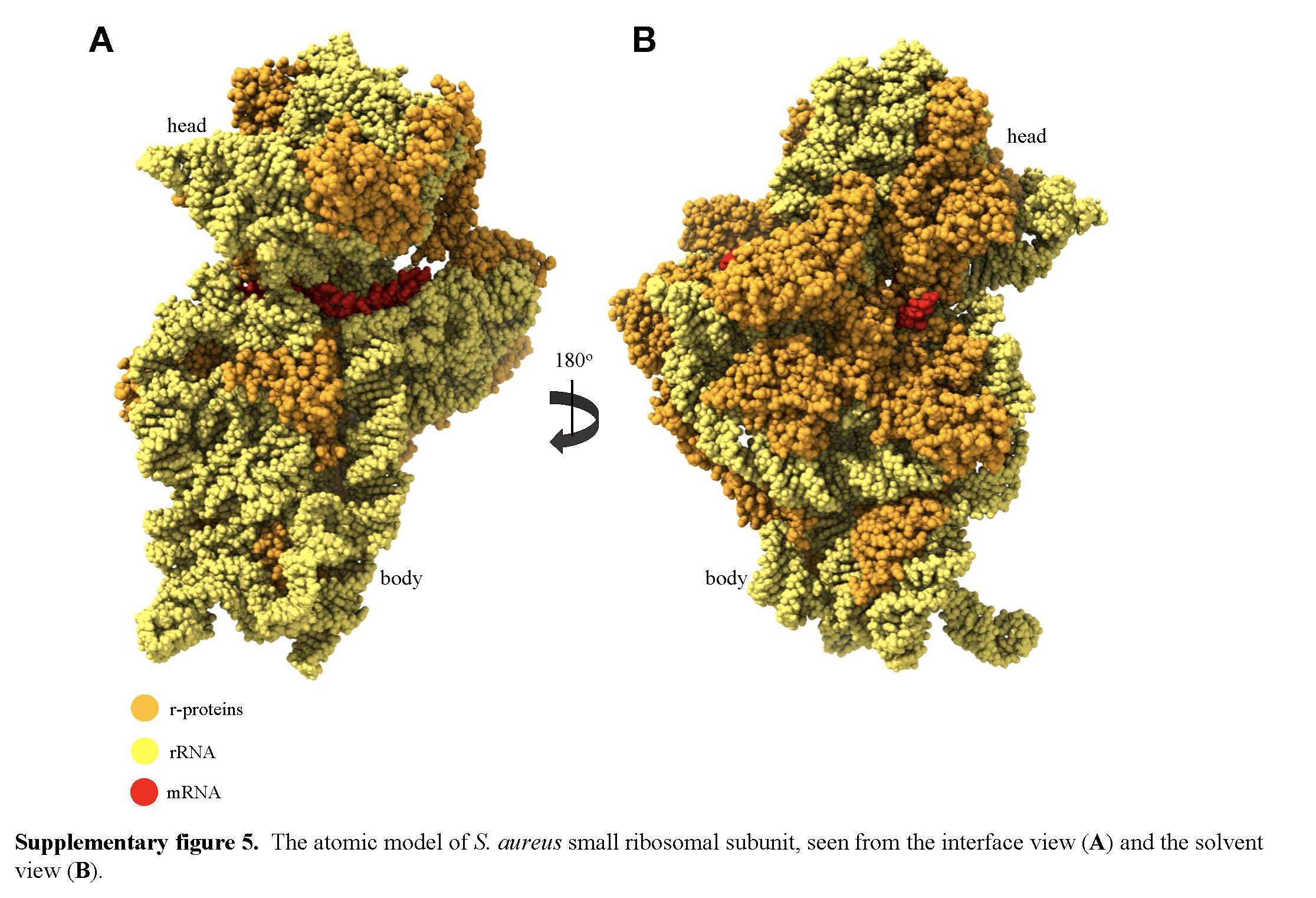

Supplement: Supplementary file 5 [file Image5.JPEG]

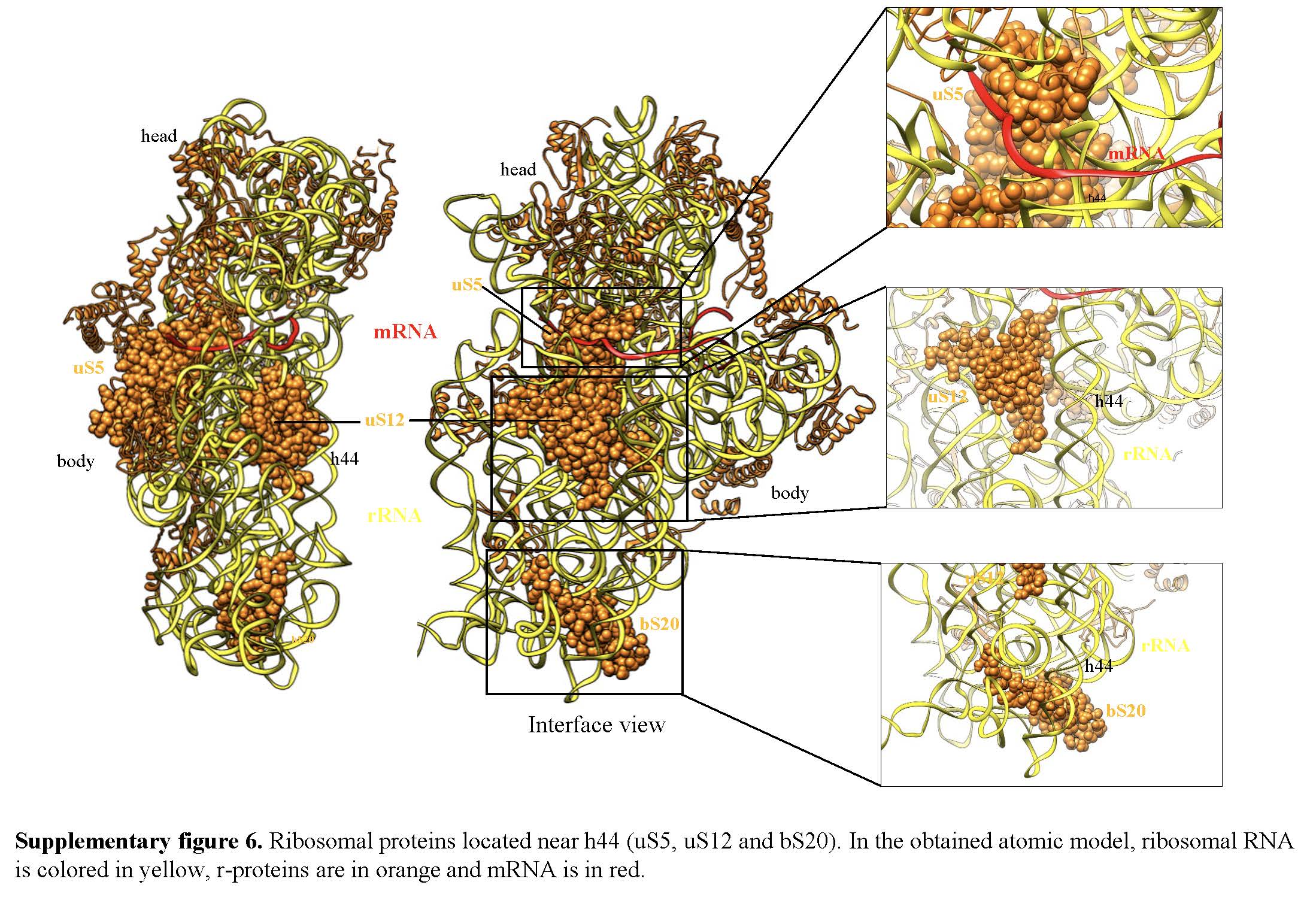

Supplement: Supplementary file 6 [file Image6.JPEG]
